# Disseminated Histoplasmosis in a Patient with Advanced HIV Disease—Lessons Learnt from Bangladesh

**DOI:** 10.3329/jhpn.v28i3.5561

**Published:** 2010-06

**Authors:** Md. Moshtaq Pervez, Brian Cobb, Nashaba Matin, Lubaba Shahrin, Evelyn R. Ford, Mark Pietroni

**Affiliations:** ^1^ ICDDR,B, GPO Box 128, Dhaka 1000, Bangladesh; ^2^ National Institutes of Health, Fogarty International Clinical Research Scholars Program, Vanderbilt University Institute for Global Health, 2525 West End Avenue, Suite 750, Nashville, TN 37203, USA

**Keywords:** Case studies, *Histoplasma capsulatum*, Histoplasmosis, HIV, Tuberculosis, Bangladesh

## Abstract

Histoplasmosis is a systemic fungal disease, also known as Darling's disease, caused by the dimorphic fungus *Histoplasma capsulatum*. It is usually self-limiting or localized in immunecompetent individuals whereas in patients with acquired immune deficiency syndrome (AIDS), it occurs in the disseminated form in 95% of cases. Although histoplasmosis predominates in the Americas (United States and Latin America, including Brazil) as an important infection among AIDS patients, it is not common in Bangladesh. In contrast, tuberculosis is extremely common in Bangladesh, with an estimated prevalence of 387 per 100,000 people. Here, a confirmed case of disseminated histoplasmosis is reported in Bangladesh in a known HIV-positive patient, which was initially suspected to be extrapulmonary tuberculosis.

## INTRODUCTION

Histoplasmosis is a granulomatous fungal disease caused by *Histoplasma capsulatum* found in soil rich in excreta of bats and birds. This disease has variable clinical features. Upper airway or digestive tract lesions are chiefly associated with systemic disease, especially affecting patients with immune suppression, as in human immunodeficiency virus (HIV) infection. Disseminated disease usually occurs in immunecompromised patients or in patients with chronic illness (1). In the setting of disseminated disease, oral lesions are present in 30–50% of patients and may occur in almost every part of the oral mucosa (2). Here, we are reporting a case of histoplasmosis manifesting with dermatologic, oral, and lymph-node lesions in the context of advanced HIV disease.

## CASE REPORT

A 32-year-old HIV-positive male, a storekeeper of a chemical company, from an eastern division of Bangladesh, was admitted to the Jagori ward (HIV inpatient unit) of the Dhaka Hospital, ICDDR,B, on 24 November 2009, with a six-month history of fever, loss of appetite, oral discomfort, weight loss (>20 kg), and multiple papular eruptions over the face and upper limbs. He had been diagnosed HIV-positive five months ago, shortly after presenting to another healthcare facility with the above symptoms and giving history of previous multiple contacts with commercial sex workers (CSWs). His CD4 count at that time was 19 cells per μL. In view of the low CD4 count, the patient was started on highly-active antiretroviral therapy (HAART) while investigations were ongoing. Subsequently, a lymph-node biopsy was performed from the left supraclavicular lymph-node, and histopathology examination demonstrated multiple lymphocytes, clusters of epitheloid histiocytes, and caseous necrotic material. The histopathological report suggested granulomatous lymphadenitis and mycobacterial tuberculosis infection as the most likely diagnosis. Considering that tuberculosis is the commonest cause of pyrexia of unknown origin (PUO) in Bangladesh and in the context of histopathological report, this patient was started on anti-tubercular therapy (ATT). However, after three months of ATT, as his clinical condition did not improve, he was referred to the Jagori ward for specialist input.

At admission, the patient was emaciated and febrile (39.6 ºC). Extensive oral thrush, multiple mouth-ulcers, and sloughing of the buccal mucosa were noted. Multiple ulcers were also noted in the nasopharynx with occasional bleeding points. A large lymph-node over anterior triangle of the left side of the neck was found which was firm, non-tender, and mobile. Dermatologic examination revealed multiple papular eruptions over the face and upper limbs, which were non-tender and non-itchy, some demonstrating spontaneous ulceration and crusting. Abdominal examination revealed mild, firm, non-tender splenomegaly. The rest of the general physical examination was normal. The examination of the cardiovascular, respiratory, and neurological system was unremarkable.

Laboratory evaluation showed low haemoglobin of 9.6 g/dL but total white cell and platelet counts were within normal limits. Venereal Disease Research Laboratory (VDRL) test was non-reactive. Liver function and renal function tests were within normal limits. Blood culture was negative. Chest X-ray was unremarkable. Endoscopy of the upper gastrointestinal tract showed candidiasis with mucosal erosion of the esophagus. Ultrasonography of the abdomen showed mild splenomegaly (3.5 cm) with no focal lesions and a normal liver.

Tuberculosis-HIV co-infection is very common but the patient's non-response to ATT suggested an alternative diagnosis. The clinical feature of papular lesions associated with a systemic illness in an HIV-infected individual was more suggestive of a disseminated fungal infection. Therefore, lymph-node biopsy from the left side of the neck was repeated. The report stated “necrotic material mixed with polymorphs, lymphocytes and histiocytes. On Gomori's Methenamine Silver stain, there are yeast-like bodies present extracellularly as well as within the histiocytes.” Histopathologists concluded that the abscess associated with deep fungal infection was morphologically consistent with histoplasmosis. A diagnosis of disseminated histoplasmosis was made, and the patient was commenced on intravenous (IV) amphotericin B (0.7 mg/kg/day), as per the Bangladesh national guideline on management of opportunistic infections (3), and his clinical condition improved dramatically within one week of treatment. Amphotericin B was continued for 21 days, and the patient was then switched over to oral itraconazole 200 mg twice daily.

## DISCUSSION

*H. capsulatum* is a dimorphic fungus, an intracellular organism, which parasitizes the reticuloendothelial system in histoplasmosis involving the spleen, liver, kidney, central nervous system, and other organs (1). The organism exists as a saprophyte in nature and has been isolated from soil, most often when contaminated with chicken-feathers or droppings. Its spores are infectious to humans by the airborne route. In 95% of the cases, infection with *Histoplasma* is asymptomatic. In immunocompetent patients, it usually manifests as self-limiting respiratory infection comprising fever, malaise, cough, and chest-pain. Most cases of histoplasmosis in patients with AIDS have been of the disseminated type. In such cases, patients present with prolonged fever, weight loss, mucocutaneous lesions, hepatosplenomegaly, lymphadenopathy, pneumonia, adrenal insufficiency, occasional intestinal ulcerative lesion, and, in some, with a sepsis-like illness (1, 4). Histopathology may demonstrate granuloma formation, as in tuberculosis and in other fungal infections; fungal stains of biopsied tissue demonstrate yeast-like structures in about half of cases (5).

In developing countries, tuberculosis is the commonest cause of granuloma formation and with suggestive clinical presentation; patients are often started on ATT as it occurred in this case. The fact that the patient worsened clinically while on ATT raised the suspicion of an alternative diagnosis.

There are important lessons to be learnt from this case, particularly for physicians caring for patients with HIV. Tuberculosis is certainly an important consideration in the context of its high prevalence in Bangladesh (6). However, in the context of HIV-associated disease, especially as the level of immunosuppression increases, it is important to consider wider array of potential opportunistic infections. Disseminated fungal infections are widely reported in HIV-infected persons and should be included in this differential diagnosis. In addition to histoplasmosis, other disseminated mycoses to consider include cryptococcosis and penicilliosis. Diagnostic confusion has been reported earlier. In one report from Guatemala (in Central America), of the eight patients who had disseminated histoplasmosis with AIDS, diagnosed over a 10-year period, all were initially admitted with diagnosis of tuberculosis with AIDS (7).

Histoplasmosis is an uncommon cause of PUO among HIV-infected patients in the Indian subcontinent where tuberculosis, especially extrapulmonary tuberculosis, is the leading cause (4). Despite a few case reports of histoplasmosis in Bangladesh (8–9), the presence of *H. capsulatum* appears common in the nearby East Indian states. A study in West Bengal, for example, showed a prevalence of skin positivity of 9.4% to histoplasmin antigen (10), and many cases of histoplasmosis have been reported nearby the Ganges river (11). There is no natural barrier or significant ecological difference between Bangladesh and West Bengal (10). Therefore, the possibility of future cases of histoplasmosis in Bangladesh should be borne in mind, particularly among medical teams caring for immunosuppressed patients. Patients with advanced HIV-associated disease are not the only population at risk for the development of this opportunistic infection; a recent report of disseminated histoplasmosis in a patient with renal transplantation returning from Bangladesh (12) is an important illustration of such a risk.

In this case, reconsideration of a diagnosis of extrapulmonary tuberculosis facilitated accurate discovery and successful management of disseminated histoplasmosis, a locally-uncommon and unexpected opportunistic infection. This serves as a timely reminder that medical teams caring for HIV-infected and other immunosuppressed patients must remain aware of conditions which may be otherwise uncommon in the environment of their patients and in the team's experience.

## ACKNOWLEDGEMENTS

The authors thank the Pathology Department of Square Hospital Ltd. for the histopathology report.
